# “Get a shot of rhythm and blues”: Songs on corona and COVID-19 vaccination

**DOI:** 10.1016/j.jvacx.2022.100155

**Published:** 2022-03-19

**Authors:** Ger T. Rijkers, Olivier Braas

**Affiliations:** Science Department, University College Roosevelt, University College Roosevelt, P.O. Box 94, 4330 AB Middelburg, the Netherlands

**Keywords:** Corona, COVID-19, Vaccination, Song lyrics, Hesitancy

In May 1956, Chess Records released the song *Roll over Beethoven* by Chuck Berry. Rolling Stone magazine, 47 years later ranked this song at position #97 of their 500 Greatest Songs of All Time [1]. Chuck Berry sings that he is suffering from “a rocking pneumonia” for which he asks for “a shot of rhythm and blues” from his doctor. The song has been covered by many, but has also been the inspiration for other songwriters including Terry Thompson. He wrote *A Shot of Rhythm and Blues* which was recorded in 1961 by Arthur Alexander, and a few years later by The Beatles in one of their sessions for the BBC radio “*Well, here’s something for you to do, get a shot of rhythm and blues*”. The “*shot of rhythm and blues*” could be signposted as the problematic metaphor in the discourse on corona and a connotation of a health promotion intervention for COVID-19 control through uptake of vaccination. Although the lyrics are dealing with vaccination, this song is not listed in the Spotify playlist of the top 25 songs on (corona) vaccines [2].

Many songs have been written about diseases, especially infectious diseases. Before the outbreak of the COVID-19 pandemic, influenza, pneumonia, and AIDS were the infectious diseases most featured in song lyrics [3]. The number 1 position of infectious diseases mentioned in song lyrics now, August 2021, has been taken by COVID-19 with 1,330 hits (https://www.lyrics.com/lyrics/COVID%2019 accessed August 28, 2021), more than twice as much as pneumonia at position 2 with 612 hits (https://www.lyrics.com/lyrics/pneumonia). The number of songs with the term “corona” in the lyrics even outnumbers “COVID-19” songs with 8,169 hits. It should be kept in mind, however, that most songs written before 2020 that contain the word “corona” are not about the circulating corona viruses but about Corona beer, cigars, or geography such as the Corona neighborhood in New York. The Paul Simon song *Me and Julio Down by the Schoolyard*, released in 1972, features a girl named Rosie, “the queen of Corona“, referring to the neighborhood in Queens, is one of the better-known examples of the latter. Nevertheless, over a 1,000 songs with “corona” as well as “virus” in the lyrics are included in the Lyrics.com database, a number which reenforces the #1 position of COVID-19 and coronavirus in the list of songs about infectious diseases.

We have performed a sentiment polarity classification of the lyrics of corona vaccines and vaccination songs published in 2020 and 2021. The word “Corona” in combination with “vaccine” or “vaccination” was found in 111 songs. “COVID-19” in combination with “vaccine” or “vaccination” was found in 97 songs. After removal of duplicates, containing both “corona” and “COVID-19”, 199 songs remained. From the lyrics, the attitude towards vaccination was classified as positive, neutral, negative, or as unknown or not applicable. In many song lyrics, vaccination was not the main theme but part of the expressed views on restrictions and quarantine measures. When the dominant sentiment in a given song was that of skepticism and sense of denial of the corona crisis, the polarity of the sentiment towards vaccination was regarded as negative. Positive attitudes towards vaccination were classified as it being the fastest and only route to get out of the crisis. The vast majority of songs also was posted on You Tube (179 out of 199), from which we retrieved the exact date of publication as well as the number of views. The data in Fig. 1 show that there was no difference between positive and negative vaccination songs in the number of views. It should be stated that the median number of views of the videos is only 207 and 66% attract less than 1,000 viewers.

**Fig. 1:**
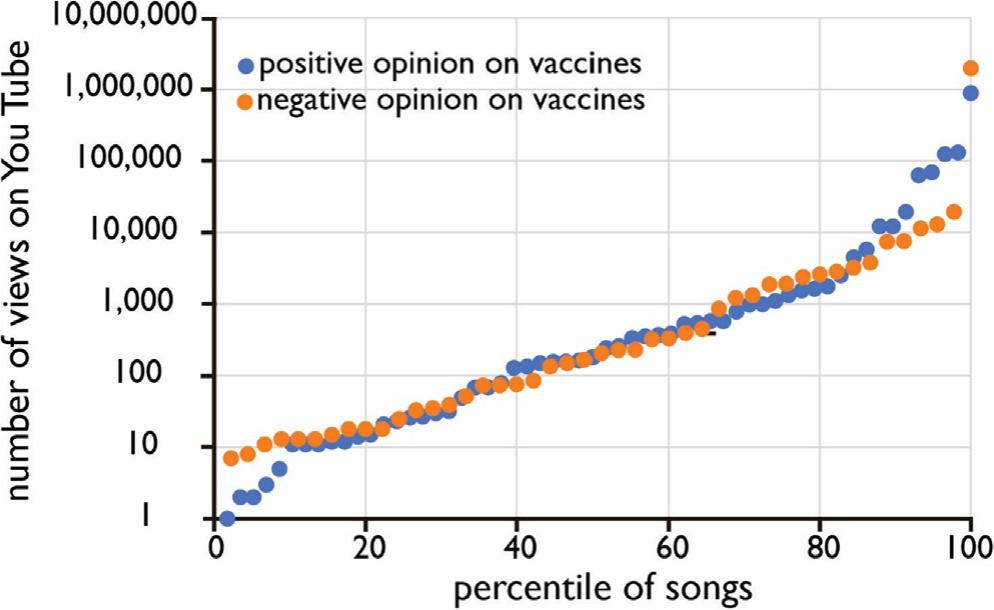
You Tube views of songs about corona vaccination (28 August 2021). Songs were retrieved from lyrics.com using the search terms “corona” or “COVID-19” in combination with “vaccine” or “vaccination” in the lyrics. Of these 199 songs, 20 were not to be found on You Tube, and of 13 others the number of views was not indicated. Of the remaining 166 songs, 58 conveyed a clear positive message and 45 a negative one. Songs were ranked based on number of You Tube views.

Many, particularly rap songs about corona and vaccination discourage to take the vaccine. Three examples are Freericky: “… they schemin' people steamin' vaccinations we done took boy!”, Mack Chamberlain: “ ….… vaccination sample size, Africans all pressed decline... “ and Kozmik Force “... by any means, avoid the vaccine... “). If the number of You Tube views would be a proxy for the popularity of these songs, the impact is rather limited. Freericky has 229 views in 16 months, Mack Chamberlain 4 views in 3 months, and Kozmik Force 1,871 views in 16 months (all accessed on August 8, 2021). These numbers are dwarfed by the video remix “*Vax That Thang Up”* from Juvenile’s 1999 original “*Back That Thang Up*”, which gained more than three million views in just one month. It therefore appears that repurposing of existing songs with vaccination related lyrics is an effective way to reach a larger audience. Examples from other styles of popular music are Neil Diamond who turned *Sweet Caroline* into a corona public service announcement (more than two million YouTube views) and Dolly Parton with her *Vaccine* version of *Jolene* (375 k views).

It must be admitted that also big mainstream artists have published material with outright corona-denial content. Van Morrison and Eric Clapton (!; exclamation mark added by GTR) recorded *Stand and Deliver*, with the lyrics “ … You let them put the fear on you, Stand and deliver, But not a word you heard was true” and Van Morrison solo in *No more lockdown*, with the lyrics “ … No more threats, no more Imperial College Scientists making up crooked facts … “. These two songs, for all the good reasons, are on position #1 and #2, respectively of the Variety Magazine Worst Songs of 2020 [4].

The current corona vaccines are effective in preventing COVID-19 disease and hospitalization, including the cases caused by infections with variants of concern. In the introductory paragraph above, the “shot of rhythm and blues” is interpreted as meaning a shot with a vaccine. That is not necessarily true, it could also be the administration of an (intravenous) drug, such as an antibiotic for the rocking pneumonia. Those who hesitate or refuse to take the corona vaccine, may need a shot of corona medicine to prevent hospitalization for COVID-19 when they become infected. The nucleoside analogue molnupiravir [5] and the protease inhibitor ritonavir [6] are oral COVID-19 drugs which could fulfill that purpose.

In a commentary in the Guardian, Brooke Harrington argues that the difficulties of trying to convince vaccine doubters and refusers can be compared with the sociology of fraud [7]. In this concept, developed by Gofman [8], the *mark*, who is the victim of an *operator*, has to be convinced to accept the inevitable by a *cooler*. The most effective *coolers* come from relevant reference groups [9]. Everyone, including vaccination doubters and refusers, belongs to a number of different, but sometimes overlapping reference groups. Family and friends, socioeconomic, religion, political, sports, news channels and newspapers, anything, including musical preference can be considered a reference group. The concept of reference groups was developed half a century ago but in the here and now, the algorithms of Google, You Tube, and especially social media play a major role in creation and enforcement of (opinions within) reference groups. In times of corona, with (physical) social distancing in place, social media would even play a more important role, especially for those who do not make use, or do not believe, traditional media such as network television and newspapers. From an analysis performed before the current pandemic, it appeared that 65% of videos on You Tube on vaccination were anti-vaccine and had a higher degree of closeness centrality than pro-vaccine videos [10]. Twitter on the other hand, also before 2020, even though neutral tweets were predominant (69%), the proportion of positive vaccine-related tweets (22%) was higher than negative (9%) [11]. Our data regarding the lyrics in You Tube music videos on corona vaccination show an overall 41% positive as compared to 30% negative attitude towards corona vaccines and vaccination. There was no significant difference in the attitude towards vaccination between songs published in 2020 (when vaccines were not available yet; n = 92) and 2021 (n = 56).

Music is used all over the world to convey information, including raising awareness about COVID-19 [12], [13], [14]. While the database we have used (Lyrics.com) is multilingual, it will be biased towards songs written in English. In our dataset, 6 songs were in Hindi of which 4 were positive about vaccination. The number of French, German, and Hungarian songs was too low to allow for a meaningful analysis. The dominance of English lyrics is a limitation of our study, making it difficult to estimate the impact for the global health promotion mission. Anti-vaccination sentiments can be rooted deeply, requiring cooling from as many reference groups as possible. Thus, listening to a vaccination-positive song from your favorite artist in your native language probably is not enough for outspoken anti-vaxxers to change their mind. It could however, in the context of signals from other reference groups, tip the balance for those still hesitant.

## Declaration of Competing Interest

The authors declare that they have no known competing financial interests or personal relationships that could have appeared to influence the work reported in this paper.
